# Health and social service provider perspectives on challenges, approaches, and recommendations for treating long COVID: a qualitative study of Canadian provider experiences

**DOI:** 10.1186/s12913-025-12590-3

**Published:** 2025-04-08

**Authors:** Anh T. P. Nguyen, Chantal F. Ski, David R. Thompson, Susan E. Abbey, Stefan Kloiber, Natasha Yasmin Sheikhan, Peter Selby, Roslyn Shields, Susan L. Rossell, Gillian Strudwick, David Castle, Lisa D. Hawke

**Affiliations:** 1 Centre for Addiction and Mental Health, Toronto, ON Canada; 2 School of Nursing and Midwifery, Queen’s University Belfast, Belfast, UK; 3 University of Toronto, Toronto, ON Canada; 4 Centre for Mental Health, University Health Network, Toronto, ON Canada; 5 Centre for Mental Health, Swinburne University of Technology, Melbourne, Australia; 6 University of Tasmania, Hobart, Australia; 7 Tasmanian Centre for Mental Health Service Innovation, Hobart, Australia

**Keywords:** Long COVID, Post COVID-19 syndrome, Service providers, Treatment recommendations, Qualitative research, Health services research

## Abstract

**Background:**

Many people who contract the SAR-CoV-2 virus present with multiple persistent and debilitating physical, cognitive and mental health symptoms that endure beyond the acute infection period. This new syndrome – generally referred to as long COVID – negatively affects patients’ emotional wellbeing and quality of life, and presents a major challenge for treatment providers. Considering the lack of evidence-based treatment and supports, this qualitative descriptive study explores the experiences of Canadian health and social service providers working with individuals with long COVID, as well as their suggestions for intervention development.

**Methods:**

Twenty health and social service providers between the ages of 29 and 57 across Canada completed virtual individual interviews to discuss their care experiences and service recommendations for long COVID. Participants were from a range of service sectors, including primary care, rehabilitation, mental health, and community support. Interviews were recorded, transcribed, and analyzed using codebook thematic analysis.

**Results:**

Four themes illustrated providers’ the experiences of (1) selecting personalized treatments based on patient presentation and similar conditions amidst uncertainty; and their recommendations for long COVID services, including (2) building an integrated and evidence-based model of care; (3) providing holistic support for patients and families through psychoeducation and daily living resources; and (4) caring for mental health in long COVID.

**Conclusions:**

Canadian health and social service providers are adopting personalized treatment approaches to address the symptom persistence of long COVID in the face of a considerable knowledge gap. A comprehensive, integrated care pathway is needed to support patients’ physical and psychosocial wellbeing while increasing provider preparedness to treat this complex condition.

**Supplementary Information:**

The online version contains supplementary material available at 10.1186/s12913-025-12590-3.

## Background

Long COVID, also known as post COVID-19 syndrome, is comprised of a set of physical, mental, and cognitive health complications, which persists for a minimum of 3 months after the onset of an acute infection with SAR-CoV-2 virus [1–3]. While most COVID-19 cases are associated with an asymptomatic presentation or brief illness, meta-analytic research suggests that the incidence of long COVID is between 10% and 30% among individuals who contract the virus, and 50–70% among those who are hospitalized [4–6]. First identified by patients [7], long COVID encompasses an extensive constellation of symptoms, ranging from fatigue, brain fog, headache, muscle pain, and cognitive deficits, to cardiovascular issues and psychiatric concerns [8, 9]. Populations more vulnerable to developing long COVID include females, racialized minorities, those who have a more severe COVID-19 infection, people who smoke, individuals living in poverty, and those with obesity or other physical or mental health comorbidities [10–13]. Although a direct connection between long COVID and other post-viral or chronic conditions has not been well demonstrated, long COVID shares numerous overlapping symptoms with the sequelae of viruses like Ebola or SAR-CoV-1, postural tachycardia syndrome (POTS), and myalgic encephalomyelitis/chronic fatigue syndrome (ME/CFS) [9, 14, 15].

In addition to complex physical symptoms, international research demonstrates impaired mental health and quality of life among some individuals with long COVID, who can experience reduced role functioning, social withdrawal, and changes to daily living [10, 11, 16–18]. Longitudinal research has reported worse mental health outcomes among individuals with long COVID, compared to those with a brief COVID-19 illness and the general public [19]. In particular, long COVID has been linked to the onset or exacerbation of several neuropsychiatric and emotional symptoms in some patients, including memory loss, insomnia, depression, anxiety, and symptoms of post-traumatic stress disorder related to the COVID-19 infection [10, 17, 18]. For some patients, long COVID can become a debilitating, multi-faceted, and prolonged disability, resulting in substantial social, psychological, and financial burden [20, 21].

However, access to treatment and support for long COVID remains limited. Several qualitative studies report obstacles for patients in seeking help, including the siloed healthcare system, the absence of long COVID-specific services, and illness invalidation by professionals [22–28]. Indeed, standardized treatments, necessary healthcare infrastructure, and social services are in their infancy as long COVID is still a new and complex clinical entity. Recent reviews of research on interventions for long COVID indicate a critical gap in the literature, with limited clinical trials under way [29–33]. Clinical practice guidelines for the treatment of long COVID have been published by the National Institute for Health and Care Excellence (NICE), which recommend a holistic, integrated and multidisciplinary approach in caring for individuals with long COVID [2]. Long COVID recovery requires a collaborated network of medical care, mental health support, rehabilitation, and social services to address the multi-systemic, long-term, and personalized needs of patients [34–38].

As research on long COVID is progressing, numerous studies have explored patient experiences [22–28, 39–46], while research on the perspectives of the service providers actively involved in long COVID care remain fewer, originating mainly from Europe and primary care settings [42–48]. Previous studies have identified several systemic challenges to providing care for long COVID, including the lack of training on diagnosis and management for general practitioners, access to specialists and continuity of care, and community service navigation [42–48]. Practitioners also recognized that limitations of the current service structure can hinder marginalized, underserved populations from accessing services [47]. However, experiences of providing care from multi-disciplinary lenses (health and social services) for individuals with long COVID in various service settings, as well as socioeconomic factors influencing services from provider perspectives, requires further research. Recommendations for future treatment and service improvement from providers also remain a gap in the literature.

### Objectives

In the present study, we qualitatively explored the experiences of working with patients with long COVID among Canadian health and social service providers from diverse disciplines, particularly their observations of long COVID as an emerging condition, challenges and current treatment approaches, along with their perspectives on equity, diversity, and inclusion in long COVID care. We also solicited providers’ recommendations for appropriate services and interventions, as well as how to develop support for patients with long COVID.

## Methods

This study is part of a larger grant funded by the Canadian Institutes of Health Research to synthesize the literature on psychosocial interventions for long COVID [30, 33] and examine the perspectives of an independent sample of patients [27, 28]. This qualitative descriptive study aims to gain further insight into service providers’ perspectives on services and treatments for individuals with long COVID. Data were collected using virtual individual interviews and analyzed using codebook thematic analysis [49]. The research was conducted with a pragmatic worldview, given its emphasis on real-world knowledge application, action-oriented collaboration, and social justice [50]. The study is reported following the Consolidated Criteria for Reporting Qualitative Research (COREQ) [51].

### Participants

Participants were eligible if they were health or social service providers and who self-identified as having worked or currently working with individuals experiencing long COVID. Participants could be from any health or social service disciplines, anywhere in Canada and had to be able to communicate in English. A total of 20 health and social service providers were recruited, including physiotherapists (*n* = 5), registered nurses (*n* = 3), occupational therapists (*n* = 3), social workers (*n* = 3), nurse practitioners (*n* = 2), psychiatrists (*n* = 2), other physicians (*n* = 1), and a peer support worker (*n* = 1). Providers worked in diverse settings, including primary care, rehabilitation clinics, mental health centres, and community organizations. No ineligible participants or dropouts were recorded.

### Procedure

Participant recruitment took place between January and May 2023 with purposive sampling. Study flyers were circulated via email among members of the research team, institutional research partners, and long COVID rehabilitation clinics across Canada. Information about the study was published on internal and external websites of the Centre for Addiction and Mental Health (CAMH), as well as advertised on institutional social media platforms. Recruitment was also supported by our team’s connections with several professional associations and Canadian long COVID support organizations. Individuals who were interested in participating contacted the research team by text, phone, or email. Each participant completed a pre-screening call to determine eligibility.

Participants then individually attended a virtual meeting to review the informed consent form with a member of the research team (ATPN), who described the research purposes and procedures in detail. After providing their written consent with a digital signature with the electronic data collection tool REDCap [52], participants completed an online demographic survey followed by the individual interview. The consent process and the interview were completed virtually, either in two separate sessions or in one session, with an estimate of 90 min in total. Both the consent form and demographic survey were hosted on a secured server at CAMH using REDCap [52]. Participants received a $50 e-gift card for their participation. They did not have a relationship with the research team prior to the commencement of the study. The study received ethical approval from the CAMH Research Ethics Board (#030–2022).

### Data collection

Individual interviews were conducted virtually by a research staff member (ATPN) and video recorded on the WebEx videoconferencing platform, which was hosted on a secure CAMH server. All participants opted to complete both the consent discussion and individual interview in one session, with the duration of interviews ranging from 28 minutes to 65 minutes (M = 47.2 minutes, SD = 10.9 minutes). The interview followed a semi-structured interview guide consisting of 13 questions regarding participants’: (1) experiences working with patients with long COVID symptoms, including treatment approaches, current challenges, and observation of patient mental health, (2) perspectives on appropriate services and support for long COVID, (3) opinions of equity, diversity, and inclusion factors affecting themselves and their patients with long COVID. Although not pilot tested, the interview guide was developed by a team of researchers (LDH, ATPN, RS, NYS, SLR, GS), including clinician scientists with healthcare service and qualitative methodology expertise (see Additional file 1). Participants were given opportunities to answer all questions during the interview, with facilitator’s careful consideration of overlapping responses and selective use of probing questions to encourage participants to elaborate on their answers. Notes were taken by the interviewer to assist with facilitating the discussion. Recordings were transcribed verbatim by a professional transcription service and validated by the first author (ATPN) for accuracy and anonymity. No missing data was found and the transcripts were not reviewed by participants.

### Data analyses

The current study employed the codebook thematic analysis method of Fereday and Muir-Cochrane, which combines both inductive and deductive approaches to analyzing qualitative data [49]. We applied constructivist principles in deriving a flexible and evolving codebook based on our research context and initial understanding of the data, then iteratively constructing themes and subthemes with deeper immersion into the transcripts and ongoing reflexive collaboration [53, 54]. Verified transcripts were uploaded, coded and analyzed using NVivo 12 by the lead data analyst [55]. After familiarization with the data, the lead data analyst (ATPN), in collaboration with the principal investigator (LDH), established a preliminary codebook and entered this into NVivo. Each transcript was subsequently reviewed in detail and coded using the coding framework. Changes or additions were added to the codebook through meetings twice a week between the analyst and the principal investigator, for purpose of theme development. Themes and subthemes were defined and continuously refined with comprehensive reflection and discussion among the research team via email communication. Quotations from participants were systematically selected to highlight key themes, and are indicated sequentially, with no identifying information. Qualitative coding was conducted alongside data collection, with consideration of saturation. We acknowledged the limitations of the concept of saturation [56], and therefore implemented several strategies to ensure the trustworthiness of the research, including commitment to reflexivity, such as self-disclosure and reflection of potential unconscious bias, in-depth engagement with data, continuous deliberation with research partners, thorough maintenance of meeting notes and audit trails, as well as transparent reporting on methodology [57].

### Positionality statement

The first author (ATPN), who was also the interviewer and lead data analyst, is a female Research Analyst with a Bachelor of Arts in Psychology. She is an immigrant to Canada with South East Asian heritage. Despite not experiencing long COVID symptoms, she has a family member who survived severe COVID-19 infection and is currently experiencing ongoing physical and mental health concerns. This project expanded the lead author’s experiences and training in conducting virtual qualitative interviews with highly-trained health care professionals. The principal investigator (LDH) is a White, female-identified Canadian scientist with research background in psychology and lived experience engagement. Despite having contracted COVID-19, she does not experience any long-term complications. Both authors are supported by a team of multidisciplinary and culturally diverse research partners, and are committed to acknowledging the impacts of their personal experiences on the research process and iterative manuscript agreement for submissions.

## Results

Table 1 presents the demographic characteristics of the participants. Participant ages ranged between 29 and 57 years (M = 40.35, SD = 7.99) with majority being women (75%) and identifying as White (60%). Most were born in Canada (70%), had attained a master degree or higher (75%), and were located within the Canadian province of Ontario (80%). Participants reported working in diverse sectors: 45% were primarily employed within the mental health and substance use sector, the remaining worked in a mainly physical health field (35%) while some self-reported working in other sectors (20%). The majority of participants (*n* = 12, 60%) reported having worked with at least 25 long COVID patients, suggesting substantial exposure to long COVID care.

**Table 1:** Demographic characteristics of health and social service provider participants

Demographic characteristics (*N* = 20)	*n*	%
Gender		
Man	5	25
Woman	15	75
Born in Canada		
Yes	14	70
No	6	30
Ethnicity		
White	12	60
Racialized	8	40
Location		
Province of Ontario	16	80
Canadian province outside of Ontario	4	20
Education		
Bachelor degree or less	5	25
Master’s degree	12	60
Medical doctorate	3	15
Primary sector		
Mental health & substance use	9	45
Physical health	7	35
Other^a^	4	20
Profession		
Occupational therapist or physiotherapist	8	40
Nursing (RN and NP)*	5	25
Medical doctor	3	15
Social worker	3	15
Peer support worker	1	5
Number of long COVID patients worked with		
Fewer than 25	8	40
25 to 50	6	30
51–100	2	10
More than 100	4	20

^*^RN registered nurse, *NP* nurse practitioner

^a^Other sectors as reported by participants: Family Practice, Occupational Therapy, Rehabilitation

Health and social service provider perspectives on long COVID were derived into four major themes. Their experiences centered on (1) selecting personalized treatments based on patient presentation and similar conditions amidst uncertainty, while their recommendations for long COVID services included (2) building an integrated and evidence-based model of care, (3) providing holistic supports for patients and families through psychoeducation and daily living resources, and (4) caring for mental health in long COVID. Each theme consists of several subthemes, as illustrated in Table 2.

**Table 2:** Themes and subthemes developed using codebook thematic analysis

Themes	Subthemes
Selecting personalized treatments based on patient presentation and similar conditions amidst uncertainty	Adopting a personalized, symptom-based approach to helping patients manage long COVID symptoms
	Uncertainty and knowledge gap as consistent challenges in the treatment of long COVID
Building an integrated and evidence-based model of care	Establishing a multidisciplinary team of service providers with care navigation
	Improving the continuity and accessibility of services
	Increasing service provider awareness and preparedness to care for long COVID patients
Providing holistic supports for patients and families through psychoeducation and daily living resources	Educating patients about long COVID symptoms and self-management
	Increasing support for maintaining daily living
	Providing support and family and caregivers
Caring for mental health in long COVID	Mental health impacts of long COVID and obstacles to emotional recovery
	Enhancing patient mental health with professional support, community building, and provider validation of long COVID

### Theme 1: selecting personalized treatments based on patient presentation and similar conditions amidst uncertainty

Participants reported relying on patient testimony and presentation, as well as their knowledge of other similar conditions, to inform their treatment approaches. Service providers described adopting a personalized, symptom-based approach to helping patients, while facing consistent challenges in understanding long COVID and in managing their patients’ symptoms.

The majority of participants agreed that individual presentation is key to care for long COVID. They recounted stepping away from the notion of one-size-fits-all treatments and focusing on each patient’s personal experience with long COVID. Service providers expressed the importance of understanding each patient’s medical and infection history, symptom constellation, functional limitations, and treatment goals to develop an individualized care plan. Many service providers reported adapting treatment plans to the patient’s needs and comfort levels, with a focus on helping them regain important functional abilities that support daily living. Person-centered approach to long COVID care is emphasized by one participant:

*So individuality is absolutely essential.[…]This is not a cookie-cutter kind of a condition*,* and therefore care planning can’t be cookie cutter.* (Participant 1)

In the face of uncertainty, participants discussed their engagement in continuing professional development to increase their knowledge of long COVID and help them select appropriate treatments. In particular, many reported reviewing the literature on past pandemics and other syndromes like POTS or ME/CFS, which have overlapping symptoms, to constantly keep themselves updated with the fast-paced research on long COVID and identify suitable treatments, as stated by one provider:

*We’re constantly learning and waiting to see if there’s anything novel or different. And then*,* like I said*,* we are just currently working with what we understand to be the disease*. (Participant 2)

Based on the existing knowledge about POTS and ME/CFS treatments and patient presentation, health and social service providers agreed that fatigue management and energy conservation are the core components of long COVID intervention. Service providers reported educating patients on managing their daily physical, emotional, and cognitive energy, as well as designing a stepped care plan that slowly increases patient activity level to help them regain independence. Participants further emphasized the importance of patients accepting their new limited capacity, introducing more frequent breaks into their days, taking quality rests, and learning to ask for help. In particular, one participant highlighted the crucial role of pacing in managing long COVID fatigue:

*You start with pacing. You have to do pacing. If you can’t do pacing*,* nothing else will work. Because that’s the bread – that’s the basics – the bread and butter of long COVID is that fatigue management. If you cannot manage fatigue*,* you can’t predict*,* you can’t function*,* and so it starts with the pacing*. (Participant 3)

Participants identified challenges that hinder their efforts in caring for patients with long COVID, which include uncertainty about symptom persistence and the knowledge gap about the condition. Many participants highlighted that long COVID is a complex condition and that it is often a challenge for providers to grasp the intricate symptom profile, prolonged functional deficits, and prognosis. From the providers’ perspectives, each patient may experience unique manifestations, resulting in limited physical and cognitive capacity that prevents them from upholding intense treatment regimens, as one participant stated:

*The very nature of the condition itself is extremely challenging.[…]In terms of being able to interpret*,* as a patient*,* what I’m capable of doing and what the likely consequences of that are going to be on my symptoms is incredibly challenging.[…]It’s the most difficult condition I’ve ever seen from a self-management perspective*,* because the symptoms appear kind of random or very*,* very challenging to interpret*. (Participant 4)

Service providers reported struggling with the lack of knowledge synthesis, standard best practice guidelines, and dissemination, especially with information specific to the Canadian healthcare system. To navigate the unknowns, participants reported tailoring interventions to their patients’ symptoms and exploring different treatment avenues, while keeping the patients safe and waiting for official recommendations. One participant described their struggles with the long COVID knowledge gap:

*On the other side of it*,* because it’s new and emerging*,* there’s no*,* as far as I’m aware*,* best practices quite yet developed as to how to properly rehabilitate somebody with long COVID. The research just hasn’t caught up yet. So it can sometimes seem a little bit*,* you know*,* tricky or*,* you know*,* not as kind of standardized as I’m used to treating other conditions.* (Participant 5)

According to the participants, the many knowledge gaps, the need to look at other conditions for guidance, and the complex presentations have left them feeling frustrated, overwhelmed, and sometimes helpless, with one provider explaining:.

*And then you feel as a service provider*,* I’m not doing enough. And I know that’s not on me. It’s on what the condition doesn’t allow us to know about. So I think that frustration is two-fold. You feel it wholeheartedly with our patients. But being the service provider to hear all of that*,* you feel it just the same as well*. (Participant 6)

### Theme 2: building an integrated and evidence-based model of care

Service providers discussed their recommendations with regard to developing appropriate and comprehensive healthcare services. Their suggestions primarily included establishing a multidisciplinary team with care navigation and increasing the quality and delivery of services, while strengthening providers’ preparedness to treat long COVID.

In particular, service providers suggested that patients need input from diverse service providers with multi-system perspectives. Participants proposed a long COVID care network consisting of well-coordinated collaborations between primary care, specialists, and community healthcare providers. One participant emphasized their support for the multidisciplinary care model in long COVID:

*So I think that an interdisciplinary model without question is the right way to go. The research is there. There’s data behind it in terms of improvement of symptom scores*,* and the functional scores and all that kind of stuff*. (Participant 1)

Providers suggested that the core of this network encompasses medical support from family physicians/general practitioners or specialists, and rehabilitation from physiotherapists or occupational therapists. Additionally, participants also referred to the essential roles of mental health clinicians and other health educators like social workers or nutritionists in building coping skills and recovery routines. The following quotation underscores the notion of an interconnected network of care providers:

*Having the complex care people come together*,* rather than having to send someone to a variety of places. So*,* having it be a more centrally functioning network*,* so that it’s a little bit easier to get things all in one place*. (Participant 7)

Several providers emphasized the values of a navigator role within this network to guide patients through the process of accessing care. They suggested the role could be filled by a case manager, nurse practitioner, social worker, or other community health worker, who is knowledgeable of available resources and biopsychosocial factors influencing health and access to healthcare, as mentioned by one participant:

*Some of the navigation to whether it’s things that make life more affordable in various ways*,* absolutely. And navigation*,* how do we get to and from appointments and things like that. Those can be barriers that I think that social workers could absolutely offer a great deal of assistance*. (Participant 1)

Many service providers also recommended that continuity of care and service accessibility be emphasized in an interdisciplinary care model. With the complex and often chronic nature of long COVID, participants proposed lengthening the course of treatment and providing opportunities for longer-term follow-ups to observe patient progress over time. Participants believed the key to effective long COVID treatment is to support the consistent application of new knowledge and new habits among patients, together with seamless transition from inpatient to outpatient services, or from hospital to community settings. They regarded the idea of increasing service accessibility through flexible delivery modes and alternative clinic hours as a means of reducing the physical, financial, and geographical barriers to accessing care. Continuity in long COVID care to ensure intervention application was reflected in a participant’s suggestion:

*I think it might be worthwhile to have*,* I guess*,* virtual follow-ups to see how patients are doing once they’re home. And to be able to provide*,* I guess*,* virtual counselling*,* or treatment. Whatever it is that way. Just to make sure that the skills*,* and the strategies that were gained*,* and were learned in an inpatient setting are still being applied*,* like*,* back at home.* (Participant 8)

Several service providers discussed the invisibility of long COVID, in which its symptoms are often under reported and under recognized, particularly with the lack of diagnostic testing evidence and the decline in public dialogue surrounding the COVID-19 pandemic. Participants acknowledged that misunderstanding and even dismissal of patient experiences with long COVID, especially by healthcare providers, can inflict a heavy burden on patients’ physical and mental well-being. One provider described the nuances of long COVID symptoms:

*But it’s very similar to a concussion. You’re not going to see evidence of it*,* like*,* in imaging. It doesn’t mean that it’s not there and that the consequence aren’t real. I think with long COVID*,* there isn’t*,* like*,* a definitive test for it but it doesn’t mean that the experience of it is any less real or that the functional impact is any less*. (Participant 9)

Many participants emphasized the importance of service providers staying informed and validating the diagnosis of long COVID. They called for the condition to be recognized as a chronic illness and a disability, especially by healthcare professionals and insurance agencies. According to the interviewees, rightful public awareness will help promote the development of specialized clinics and the provision of resources for individuals suffering from long COVID. One participant advocated for a better understanding and acknowledgement of the condition in the absence of normal testing evidence:

*What I always emphasize is just the importance of people in the health field*,* getting knowledgeable and looking at what they’re dealing with. There’s something real happening here*,* just because we can’t see it with our regular tests*,* doesn’t mean it’s not happening. It’s just our tests aren’t very good*. (Participant 10)

Service providers indicated that they require enhanced professional development. They proposed an increase in interdisciplinary knowledge exchange among providers, through forms of workshops, conferences, or newsletters. Participants encouraged providers with experience in developing long COVID clinics to share their expertise, especially with regard to improving the direct linkage between medical professionals with allied health professionals. One participant highlighted the importance of knowledge sharing with primary care professionals:

*I think definitely dissemination of best practice like development and then dissemination to primary care providers and perhaps to emergency departments. Like the staff in an emergency department to support the care of these patients would be helpful because then it would not only legitimize the diagnosis*,* so patients could then go to a provider and not feel as dismissed or invalidated. So I think that that’s an important part*. (Participant 11)

Participants also discussed challenges with service funding, human resources, and worker mental health. They called for more support to expand and sustain long COVID clinic models, guarantee adequate workforces, and enhance provider resilience. For instance, one provider shared their thoughts on maintaining their long COVID clinic when discussing the need of more resources:

*I think this actual clinic is very– there isn’t necessarily longevity in it. We don’t know whether or not this program is going to be here in six months in a year. So there’s a lot of things that you may want to do as a clinic in terms of advocacy*,* in terms of building more programs*,* building more within the modules. But if you’re constrained by not knowing how long this program is going to be existing*,* that is really hard to do*. (Participant 6)

Another participant highlighted the mental burden and support for service providers:

*I think it also is emotionally draining and can touch close to home. […] For people*,* especially seeing some of the most severe long COVID cases and then worrying for themselves*,* so I think that’s also important to highlight. And for anyone who’s seeing mental health patients*,* to ensure that they have their own coping strategies and support*. (Participant 12)

### Theme 3: providing holistic supports for patients and families through psychoeducation and daily living resources

Service providers highlighted the importance of holistic support for patients, by promoting up-to-date and evidence-based long COVID psychoeducation and self-management strategies, as well as providing daily living resources for patients and family members. Specifically, participants advocated for educating patients about the nature of long COVID syndrome, common symptoms, and its impacts on patient physical and mental health as a key point during any clinical visit. They emphasized the need to inform patients of available resources and set realistic expectations about treatment and rehabilitation progress. Participants acknowledged that transparent communication with patients can help reduce confusion and fear about emerging and persistent symptoms, validate their experiences, and establish a growth mindset for recovery. One participant suggested several ideas on how to inform patients about long COVID:

*It doesn’t even have to be like a group program. It could be like a video that they’d be asked to attend. So*,* some sort of like wholesome like full-some information on what long COVID is*,* how it can impact you. Like*,* some sort of resource like that*,* like a written resource document or something on just like psychoeducation on really what it is and how it can impact you and then with some sort of information where you can go for support.* (Participant 13)

Many providers agreed on the active promotion of self-management for patients as the core foundation for recovery, including the management of physical, cognitive, and mental health. They recommended that patients learn about the pillars of fatigue management, including conserving daily energy, cultivating recovery habits, and applying coping strategies. Participants emphasized energy management, as well as helping patients understand their new limits to daily activity and the importance of mindset, rest, and avoiding over-exertion. Building healthy daily habits such as attaining good quality sleep, maintaining a nutritious diet, and exercising regularly were also mentioned during the interviews. Providers suggested the important role of cultivating effective coping strategies including mindfulness meditation, breath work, stress management, acceptance and commitment. Participants believed that helping patients apply these strategies consistently will promote a sense of empowerment and trust that motivates them to overcome the challenges of long COVID. For example, one provider explained their approaches:

*What we’ve found very helpful is first establishing good recovery hygiene*,* is what we call it*,* or good recovery habits*,* which is to say*,* you know*,* if you’re trying to recover from something*,* you need to have nutrition*,* sleep*,* hydration*,* activity recovery balance*. (Participant 4)

Participants recommended that supports be built to help long COVID patients maintain their day-to-day living. They described that support for daily activities, for instance, cleaning, driving, shopping, become increasingly essential for individuals suffering from significant long COVID symptoms. Service providers acknowledged that work disruption due to reduced functioning can exaggerate financial burdens for many individuals; they stressed how a lack of resources can hinder patients from accessing healthcare or progressing with treatment. Service providers strongly believed in enhancing psychosocial support and social assistance programs to help patients maintain their daily living while overcoming the challenges of long COVID, as stated by one provider:

*It increases their fear and anxiety if they can’t manage their day-to-day life outside of the hospital. So a lot of funding is required for a lot of these patients who have been struggling with long-term COVID*. (Participant 2)

Many participants recommended extending available resources to support family members and caregivers of individuals with long COVID. Service providers believed that family and caregivers can benefit from psychoeducation about long COVID, increased knowledge of resources and strategies to build a supportive environment for patients with long COVID. This recommendation is exemplified by the following statement:

*I think it’s also very important to involve family support*,* support services that they have within their family to share and*,* you know*,* how to educate them to be able to further support that client*. (Participant 14)

### Theme 4: caring for mental health in long COVID

From participants’ perspectives, mental health is an important aspect of long COVID care. Many service providers observed an increase in mental health concerns and discussed barriers hindering patient mental wellbeing. They also suggested strategies to help build resilience among patients.

Participant observed significant impacts of long COVID on patients’ mental health and emotional wellbeing, such as increases in anxiety, depression, frustration, distress, and symptoms of post-traumatic stress disorder. They noted that, while some patients were able to cope without professional interventions, a large proportion of individuals with long COVID experienced more severe and debilitating mental health and emotional concerns which prevented them from engaging in treatments. Providers understood that many patients were confused about their health status, frustrated with the healthcare system, and worried about their future, and these altogether increased their anxiety level. Participants also noticed a persistent sense of grief, potentially over the loss of role functioning, independence, social engagement and the changes in their self-identity due to long COVID. One provider acknowledged the impacts of long COVID on patient mental health:

*I think you can see it’s did a number on some people. Kind of like low mood*,* like*,* sometimes you’ll see some of them where they’re very flat. They don’t want to participate in much and not many things are motivating them*. (Participant 15)

Furthermore, participants identified numerous barriers to improving patient’s mental wellbeing while dealing with long COVID, including limited functional capacity, lack of social support and resources, as well as challenges due to pre-existing mental health concerns, as explained by one participant:

*We’ve got people who are newly disabled with a chronic illness*,* not able to fulfill life roles*,* not able to work*,* experiencing financial distress. Then we’ve got them not experiencing support from their family*,* friends*,* and most importantly*,* their healthcare team. So*,* a lot of them are going through being gaslit about their symptoms*,* and not having a lot of support. So*,* that definitely adds to mental health strains*. (Participant 10)

Service providers accentuated the need to incorporate mental health-specific interventions in treatment planning. In particular, they endorsed psychotherapy or counseling with mental health professionals to help patients process the changes due to long COVID. Service providers suggested supporting patients by helping to reframe their mindset, increase acceptance, help them focus on the present, and support them in maintaining hope. They also highlighted the value of teaching patients coping mechanisms for stress management, emotional regulation, and self-care with psychotherapeutic tools such as cognitive-behavioral therapy or mindfulness.

*And then*,* I think access to psychotherapy is always a problem*,* but I think incredibly useful in helping individuals process and work through their challenges and have actionable strategies that they can implement*,* in terms of kind of managing symptoms and also increasing resilience in coping*. (Participant 12)

Participants also highlighted the importance of support groups or peer support programs as an additional network of care. Participants believed that support groups could help normalize the experience of long COVID, build a sense of community through empathetic human connections, and establish a platform for advocacy with increased awareness, for example:

*Maybe having some meetings where they can go to*,* like groups. […] Even just not for the long term*,* but just like for COVID*,* for people that want to talk about it*,* because it’s a lot to come off your chest sometimes. And like that’s going to be the beginning steps to get people back to normalcy*,* is to be able to talk about it and then realize that they’re not the only one.* (Participant 16)

Many participants emphasized the importance of applying effective clinical skills and building a strong therapeutic alliance with patients, even in physical healthcare services, to promote mental health and wellness in addition to referring patients to mental health resources. Acknowledging that medical visits can be a source of anxiety for patients, service providers outlined the first important step in establishing rapport is to practice open-mindedness and active listening, allowing patients time and space to communicate their symptoms. They also urged health and social service providers to adopt a trauma-informed approach, to understand the biopsychosocial barriers for patient recovery, and to provide patients with empathy and validation of their experiences. Participants further suggested other providers find a common language and work collaboratively with patients, helping them build a sense of control over their physical and mental health following a strength-based, empowerment-oriented perspective. One participant emphasized the importance of building therapeutic relationships with patients using trauma-informed lenses:

*So I think there’s a huge impact on patients*,* in terms of being able to see themselves in their service providers and to trust their service providers*,* based on their own personal experiences. I think experiences of trauma for example*,* in the past whether that be due to sexual trauma*,* or discrimination*,* or intergenerational trauma– there’s lots of reasons I think that impact a patient’s trust in healthcare. And so*,* I think being open to that*,* understanding that*,* and accommodating that*,* is important.* (Participant 12)

To summarize, participants in our study emphasized a need for mental health interventions, peer support, and strong therapeutic alliances to address the complex physical and emotional challenges faced by individuals with long COVID.

## Discussion

The present study explored the current situation and future directions of healthcare for long COVID through the lenses of health and social service providers across disciplines, about three years after the start of the pandemic. Four major themes were generated, describing the providers’ experiences and perspectives on interventions for long COVID. As Canadian service providers are (1) selecting personalized treatments based on patient presentation and similar conditions amidst uncertainty, they recommended (2) building an integrated and evidence-based model of care (3), providing holistic supports for patients and families through psychoeducation and daily living resources, and called for more effort in (4) caring for mental health in long COVID. As long COVID research is in its early stage and resources remain limited, health and social service providers were using a symptom-based, gradual treatment approach to help patients manage long COVID. Acknowledging the significant impacts on patient daily functioning and mental health, participants suggested more holistic support for self-management, everyday activities, and psychological wellbeing. They also emphasized the importance of support for family and caregivers of people with long COVID.

Providers experienced the challenge of limited knowledge about assessments and treatments, while they have the desire for and expectation of structured, precise guidelines and integrated care pathways. Previous research on patient experiences of long COVID has reported inconsistency and unavailability of healthcare services, in addition to long wait times and lack of access to primary care, which exacerbate the physical and emotional burden on patients [22–25]. In line with our findings, several qualitative studies on the perspectives of healthcare professionals in the United Kingdom acknowledged the lack of information to support diagnosis and management for providers, especially with regard to recognizing the onset of the condition and identifying appropriate treatments [42–44, 46, 47]. Consistent with the current literature, our study highlighted the critical gap in standardized knowledge and interventions for long COVID within Canadian healthcare systems. Similar to our UK counterparts, Canadian service providers resorted to the literature on similar conditions such as POTS and ME/CFS to support patients, while struggling with the persistence of long COVID symptoms and systemic barriers to service delivery.

To address these challenges, participants called for the development of a comprehensive, evidence-based network of care for long COVID that integrates interdisciplinary expertise and service coordination. This aligns with other research on long COVID service development and implementation, as well as published clinical practice guidelines [2, 23, 25, 26, 47]. An investigation into the perspectives of a unique set of healthcare professionals who also experienced long COVID first hand proposed a holistic, multi-leveled, and co-designed service structure embedded in primary care with potential for appropriate specialist referrals and community follow-up [26]. Similarly, a scoping review echoed the need for such a well-defined, integrated healthcare pathway that encompasses medical, rehabilitation, and mental health services and utilizes specific treatment approaches designed for long COVID [36]. Recommendations from participants in our study echoed the development of multidisciplinary collaboration while emphasizing continuity of care, public recognition, as well as support for providers and caregivers.

Furthermore, our findings revealed the desire for more coherent, inclusive, and consistent standards of care for individuals with long COVID. Service quality principles established by individuals with long COVID in the UK emphasized the need for providers to support patients in navigating the limited and fragmented healthcare system, while maintaining clear clinical boundaries [25]. In our study, service providers proposed implementing a navigator role as a key provider in the long COVID healthcare network in order to mitigate the navigational burden and systemic challenges in accessing care for patients. Strong clinical skills such as open-mindedness, active listening, and validation are also considered by participants as a key part in building quality service experiences and mental wellbeing for patients. As the lived experience literature on long COVID has revealed that illness invalidation and discrimination against patients are common [23, 24, 27], providers from our study deemed that service providers adopt a trauma-informed, person-centered approach to increase equitable care and lessen the emotional repercussions of inadequate care on patients and caregivers.

The paucity of services for and understanding of long COVID underlines the urgent need to enhance awareness of this emerging syndrome among the public and particularly within the healthcare community. A recent critical review of the literature has given prominence to a comprehensive and robust research agenda on the biomarkers, mechanisms for these neuropathologies, and potential interventions for long COVID symptoms, with the inclusion of under-represented groups and the appropriate engagement of people with lived experience in research processes [9]. Professional training and knowledge dissemination for healthcare providers, insurance agencies, social support organizations, and those involved in long COVID care demand urgent attention. It is important to address the critical knowledge and service gaps based on empirical evidence and pre-existing literature on other post-viral syndromes and associated conditions, with a focus on sustainable care and self-management [9, 40]. As our previous reviews have identified significant literature gap in mental health support for long COVID [29, 30, 33], we recommend that future research explore interventions and services which address not only the physical, but also psychosocial aspects of long COVID experiences, using cross-sectional and longitudinal designs. Attention to diverse populations and people with pre-existing conditions is also warranted.

The present study has several strengths and limitations. To our knowledge, this is the first qualitative study focusing on service providers who are providing treatment and care for long COVID patients in Canada, adding to current literature. We recruited a relatively diverse sample of Canadian health and social service providers who are involved in long COVID care. The participants varied not only by sociodemographic characteristics, but also by occupations and work settings, providing multi-facetted perspectives. Furthermore, individual interviews, as a standard qualitative data collection method, provided flexibility for participants and fostered a confidential and secure environment for them to share their clinical experiences with long COVID. However, participants were mainly from large urban areas, and the experiences of service providers in rural or remote communities are therefore not represented. Additionally, the diversity in professional backgrounds may have limited the depth of discipline-specific perspectives as participant responses were dependent of their current practices, work settings, and the number of individuals with long COVID in their care. Our sample also largely consisted of white, highly educated, female practitioners, which may have limited our findings of equity, diversity, and inclusion influencers on long COVID treatment. Future research may consider perspectives of providers from more remote, underserved areas, with more gender, racial, and educational diversity to capture a well-rounded picture of service accessibility. Lastly, as the literature on long COVID interventions is quickly progressing, the experiences of service providers may also change rapidly over time. Our study results therefore should be contextualized in light of the latest updates in the long COVID literature.

## Conclusions

This study demonstrates the ongoing challenges facing Canadian health and social service providers as they treat individuals with long COVID. Our findings emphasize the importance of developing real-world service models that provide solutions for people with Long COVID by incorporating service navigation, continuity, and accessibility in building a more integrated, cohesive and interdisciplinary model of care, as well as improving knowledge and preparedness for service providers in order to adequately address the complexity of patients’ physical and mental health needs. These findings, from Canadian health and social provider perspectives, emphasize the importance of scientific investigation and intervention planning for long COVID and the engagement with multiple stakeholders, including service users and providers, to design, implement, and evaluate a coherent and comprehensive network of care.

## Supplementary Information

Supplementary Material 1.

## Data Availability

The full dataset is not available for public review and use due to privacy and confidentiality reasons. Parts of the data generated and analysed during this study are included in this manuscript as quotations. Please contact the corresponding author for any request regarding the study data.
