# Supplementary material for: Health and social service provider perspectives on challenges, approaches, and recommendations for treating long COVID: a qualitative study of Canadian provider experiences
Source: BMC Health Serv Res. 2025 Apr 8;25:509. doi: 10.1186/s12913-025-12590-3 (PMC11977920; doi:10.1186/s12913-025-12590-3)
Supplement: Supplementary file 1 — Supplementary Material 1. [file 12913_2025_12590_MOESM1_ESM.docx]

**Long COVID and mental health: Semi-structured interview guide**

Service providers (1-1 interview)

**Preamble**

You are here today because we want to hear the perspectives of service providers, who are providing care for patients experiencing long-term symptoms after having a COVID-19 infection, which has been called ‘long COVID’.

We’re here today to discuss your experience working with patients with long COVID: the way you help these patients as well as the challenges you’ve encountered. We also want to talk about your experience with patients living with COVID and pre-existing mental health challenges. Throughout today conversation, whenever we talk about mental health, we mean both emotion, mood, energy, feeling, mental well-being as well as the use of substance such as alcohol, cannabis, or illicit drugs.

Then, we’re going to talk about what kind of services/ interventions that might be helpful for patients with long COVID. We will ask you to brainstorm the types of services that you think would be most helpful in building patient mental health and managing their condition.. This could be any type of service – feel free to get creative.

We’re also going to talk about diversity factors such as age, gender, race, socioeconomic backgrounds – like any personal factors and unique characteristics, which have influenced your experience with long COVID patients, and how we can tailor future services to these differences.

We’re going to copy our questions into the chat box. You can also use the chat box to contribute to the discussion if you prefer. We will read these comments out

Before going into the discussion, do you have any questions?

**Part 1: Experience helping long COVID patient**

1. Can you tell us about your experience working with patients with long COVID so far?
   1. Probe: How is your experience providing assessment for long COVID?
   2. Probe: How is your experience providing care or services for patients with long COVID?
   3. Probe: What about virtual care? Does that have any impact on your service?
2. To what extent do you think long COVID is a real condition?
   1. Probe: How do you recognize that a patient is experiencing long COVID?
   2. Probe: Do you make an official diagnosis of long COVID for these patients?
   3. Probe: What about your colleagues and other physicians in your clinic? Do they believe in long COVID?
3. From your observation, how has long COVID affected your patients?
   1. Probes: Their everyday activities? Their mental health? Their substance use?
4. You may have worked with patients who have experienced mental health challenges before long COVID, or some who are experience new mental health challenges. How are these groups experiencing long COVID differently?
   1. Probes: differences in their presentations? Clinical needs? Available resources?
   2. Probe: What are the differences in your treatment for these two groups?

Probe: Are there any difference among different genders?

1. How have you helped your patients cope with long COVID?
   1. Probes: making referrals, additional skills, resources, services, medication, mental health/substance use support, involving other service providers or family?
   2. Probe: How is this different from the way you work with other patients?
2. What has made it harder to help your patients to cope with long COVID?
3. Probe: clinical presentation, restrictions, services, communications, medications, missing skills or resources, personal or social barriers like income
4. Probe: What is the difference compared to other patients?
5. What are the best things or the hardest things about working with patients with long COVID?
   1. Probe: How has this affected you and the care you provide?
   2. Probe: What has worked for your patients, and for you?
   3. Probe: What has not worked?

**Part 2: Services**

Now, we’re going to ask you to brainstorm what you think would be the best possible services to help people with long COVID, particularly their mental health. We’re interested in anything that might help with symptoms, mental health and any substance use concerns. You might consider services that can help build resilience and manage one’s condition.

Take a moment now to think about what kind of supports your long COVID patients want for mental health, substance use, and wellness. You may also think about how can service providers like you best provide these services?

There are some questions to guide you when coming up with these supports.

Who would provide the services? What kinds of supports would there be for patient and their family? What kind of information or skills would be provided? How often would can patient receive services? How would the patient access services (e.g., individual vs group)? Who else would be involved?

You can add anything else that you think might help you live as well as you can – feel free to get creative!

Facilitator guide:

1. Constant probes for all the items above: who, what, when, where. Direct the conversation to mental health or integrated services.
2. Probes
   1. What about mental health? What about substance use? Would there be any mental health professionals involved?
   2. What else can we integrate in these services? What you think could have helped your patients with their mental health and long COVID symptoms?
   3. What support should there be for your patients and your family?
3. Is there anything else you would add to that?
   1. Probes: peer support, technology, self-management & resilience building
4. What would be the most important and should be prioritized?
5. How would this service help patients cope with long COVID?

**Part 3: Equity, diversity, and inclusion factors**

We think it’s important to consider how we’re all different, in terms of our age, gender, sexual orientation, ethnic and cultural background. We may also be different in our language, our religion, economic situation, and other social factors. We’re interested in understanding how these differences may have impacted your experience working with long COVID patients.

Probes: age, gender, ethnic and cultural background, language, where you’re from, your economic situation

1. Thinking about these differences, what do you think may affect or have affected your practice with patients with long COVID?
   1. Probes: What about your personal differences? What about your patients’?
   2. Probe: What about racism or discrimination?
   3. Probe: How has that influenced your experience working with long COVID patients?
2. How can services for long COVID be specialized or adapted based on your personal differences or your patients’ differences?
   1. Probe: individual vs group service?

***Conclusions***

1. Is there anything else about your experience with long COVID patients or service ideas that you’d like to tell us today?
